# Physiotherapy Strategies in Hypokalemic Periodic Paralysis: A Case Report

**DOI:** 10.7759/cureus.52294

**Published:** 2024-01-15

**Authors:** Gunjan S Ambalkar, Neha Arya, Grisha Ratnani, Raghumahanti Raghuveer, Akshaya Saklecha

**Affiliations:** 1 Neurophysiotherapy, Ravi Nair Physiotherapy College, Datta Meghe Institute of Higher Education and Research, Wardha, IND

**Keywords:** neurophysiotherapy, case report, physiotherapy, balance, physiotherapy strategy, muscle weakness, hypokalemic periodic paralysis

## Abstract

The rare neuromuscular disease known as hypokalemic periodic paralysis (hypoKPP), which results in severe muscle weakness in the extremities, is brought on by abnormalities in potassium transport within cells. Laboratory testing is confirmatory, which reveals notably low potassium levels, causing paralysis, which improves once the low potassium is restored. The patient generally complains of muscle weakness with difficulty in performing activities of daily living and impaired participation in functional tasks, with few suffering from coexisting sensory impairments. Physiotherapy generally plays a symptomatic role with motion exercises for the affected muscle groups. There is no standardized physiotherapy protocol for disease-specific impairments. A 46-year-old man complained of bilateral upper and lower limb muscular weakness and was admitted to the neurology ward. The patient also complained of having tingling numbness throughout their entire limbs and had experienced similar episodes of symptoms six months prior. During laboratory evaluation, a significantly low potassium level was found, leading to a diagnosis of hypoKPP. Following medical management, neurophysiotherapy was initiated. Physiotherapy strategy shows significant improvement in muscular strength and functional activities. Thus, this case report concludes that physiotherapy plays a vital role in managing hypoKPP by enhancing muscular strength, functional activities, and quality of life.

## Introduction

Hypokalemic periodic paralysis (hypoKPP) is a rare neuromuscular disease characterized by intermittent episodes of flaccid muscle weakness brought on by mutations in voltage-gated ion channels, which are typically mediated by potassium but are less frequently mediated by calcium or sodium. The disease is primarily associated with hypokalemia, which is primarily caused by high-carb meals or rest after exercise. Adolescents with the illness usually experience mild to severe muscle weakness that lasts for several hours or even days. Usually, when serum potassium levels return to normal, weakness goes away [1]. Most cases of hypoKPP are inherited or familial. Furthermore, acquired instances of hypoKPP have been discovered and connected to hyperthyroidism [2]. With a prevalence estimated at one in 100,000, hypoKPP is generally regarded as a rare disorder [3,4]. Most familial cases are caused by autosomal dominant inheritance with incomplete penetrance. Women often have fewer episodes of muscle weakness than men do because they are less penetrated and attack-prone than men, which results in a lower clinical manifestation of this disorder in women [5]. Attack frequency generally declines with age [6]. Theories suggest that triggering factors raise insulin or plasma epinephrine levels, which shift potassium intracellularly and lower serum potassium levels, thereby initiating the weakness episode [6]. Catecholamines such as dopamine, noradrenaline, and adrenaline can result in hypokalemia. It can also be brought on by a variety of medications, including laxatives, antibiotics, glucocorticoids, diuretics, and beta-2 agonists like salbutamol and thiazides. Furthermore, insulin injections or activating the sodium-potassium (Na+, K+) pumps, as demonstrated after exercise, can stimulate transcellular potassium, which is transported from serum into the muscles [7].

Westphall disease, hypoKPP, primary hypoKPP, and familial hypoKPP are some other names for this condition [8]. Andersen-Tawil syndrome (ATS), hypokalemic periodic paralysis (hypoPP), and hyperkalemic periodic paralysis (hyperPP) are the three subtypes of the disorder that have been identified [9]. All forms of this disease share the same underlying mechanism, independent of mutation: prolonged sarcolemma depolarization that results in sodium ion channel inactivation, which blocks action potentials and excites muscle fibres electrically [10]. Acetazolamide, an inhibitor of carbonic anhydrase, and potassium replacement therapy are the cornerstones of treatment for hypoKPP. Patients with acute flaccid paralysis typically do not consider hypokalemic paralysis [11]. Paralytic attacks are characterized by flaccidity, which is more pronounced proximally than distally, and normal to diminished deep tendon reflexes (DTRs). Before ending on their own, the episodes start out slowly and last anywhere from a few minutes to several hours [12]. Since there is no established physiotherapy regimen for impairments specific to a given disease, we attempted to manage hypoKPP by improving muscular strength and functional activities [13].

## Case presentation

Patient information 

A 46-year-old male patient came to the neurology department without a previous personal or family history. He claimed that physical activity associated with working on the farm had exacerbated his severe bilateral muscular weakness in both the upper and lower limbs (UL and LL). Later on, the symptoms got aggravated day by day, and there was difficulty in standing and walking. The patient reported that a few months prior, he experienced similar episodes of mild LL weakness. The patient was on steroid injections at the time to manage his pain. He used to ignore it at the time since it would eventually go away on its own. He was conscious, well-oriented, and aware during the examination. The patient was experiencing distress as a result of his paralysis. A physical examination was performed, and vital signs were stable. All of the sensations were intact. On motor examination, weakness of both UL and LL was seen. There was no weakness or numbness in the patient's facial muscles, and their extraocular muscle movements were normal. The response for all DTRs was normal.

In the initial lab tests, serum potassium and magnesium levels were marginally lower; they showed 1.7 mmol/L of hypokalemia without changes in calcium, sodium, or chloride, and hypoKPP was diagnosed. As replacement therapy, he received intravenous (IV) potassium, normal saline solution, and 25 ug of levothyroxine (T4) daily. His levels of potassium (3.7 mmol/L) were normal by the third day of the treatment regimen. Measures of creatine phosphokinase (CPK) and sodium levels were also performed. After conducting the nerve conduction velocity test, the outcomes fell within expected ranges. Bradycardia (heart rate 57 bpm) and hypokalemia-compatible symptoms (QT interval pseudo-prolongation, prominent U waves, and ST-segment descent) were marked on the electrocardiogram. He received IV potassium, IV sodium bicarbonate, and T4 treatments during the start of a physiotherapy session. He was also referred to neurophysiotherapy. Table 1 depicts examination findings for manual muscle testing (MMT).

**Table 1:** Physiotherapy interventions for hypoKPP hypoKPP: hypokalemic periodic paralysis; UL: upper limb; LL: lower limb; SLR: straight leg raise; PNF: proprioceptive neuromuscular facilitation

Goals	Strategies	Dosage
Patient education	It was necessary to educate the patient and his family about diseases and their effects so that they would be prepared to handle the issue in the future	Patient counselling and daily updates about progression
To improve respiratory function	Deep breathing exercises. Dyspnea-relieving position	10 repetitions x 2 sets
To reduce fatigue [14]	Pursed lip breathing	10 repetitions x 2 sets
To improve the range and muscle strength of the UL	PNF combinations of isotonic and dynamic reversals	10 repetitions x 2 sets
	Progression PNF combinations of isotonic to slow reversal	
To improve sitting balance	Bedside sitting	10 minutes
To improve range and muscle strength of UL and LL	UL strengthening with half-liter water bottle; SLR with holds	10 repetitions x 1 set with five-second hold
	Pelvic bridging	
	Strengthening exercises for both UL and LL with 1 kg weight cuff	
Sit-to-stand transfer	LL muscles are strengthened	5 repetitions x 1 set
To improve balance and gait [15]	Gait training	2 minutes
	Tandem standing and walking. Walking over and around obstacles	

Physiotherapy intervention 

**Figure 1:**
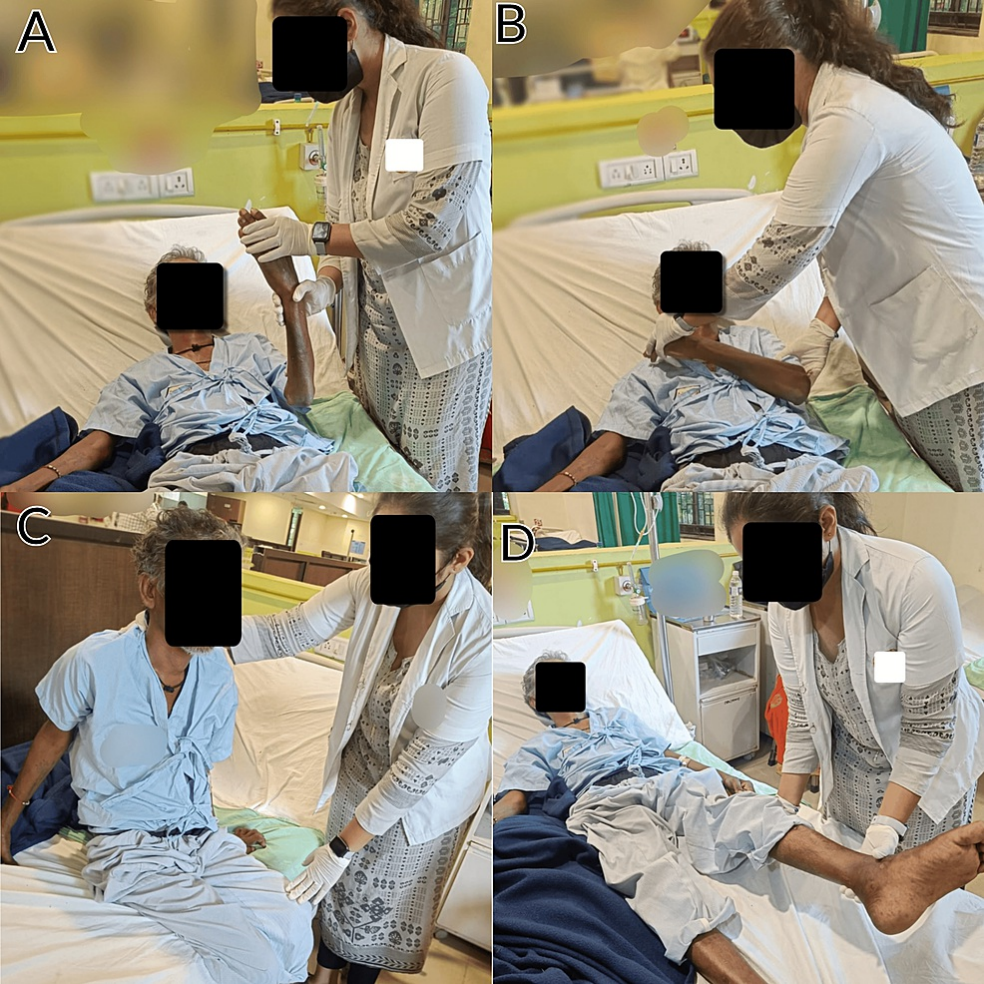
Patient performing exercises A: Strengthening of the upper limb. B: Proprioceptive neuromuscular facilitation technique. C: Bedside sitting. D: Straight leg raising

The patient was referred to the neurophysiotherapy department, where a planned intervention was designed as mentioned in Table 1. Figure 1 also represents the patient performing exercises. 

Outcome measures

Post rehabilitation, the patient showed remarkable improvement. MMT pre and post rehabilitation mentioned in Table 2 and Table 3 represent the findings of outcome measures.

**Table 2:** MMT findings pre and post rehabilitation MMT: manual muscle testing

Muscle groups	Pre rehabilitation		Post rehabilitation	
	Right	Left	Right	Left
Shoulder flexors	2/5	2/5	4/5	4/5
Shoulder extensor	3/5	3/5	5/5	5/5
Shoulder adductors	2/5	2/5	4/5	4/5
Shoulder abductors	2/5	2/5	4/5	4/5
Elbow flexors	3/5	3/5	5/5	4/5
Elbow extensors	2/5	2/5	4/5	4/5
Hip flexors	2/5	2/5	4/5	4/5
Hip extensors	2/5	2/5	4/5	4/5
Hip abductors	2/5	2/5	4/5	4/5
Hip adductors	2/5	2/5	4/5	4/5
Knee flexors	2/5	2/5	4/5	4/5
Knee extensors	2/5	2/5	4/5	4/5
Ankle plantar flexion	2/5	2/5	4/5	4/5
Ankle dorsiflexion	2/5	2/5	4/5	4/5

**Table 3:** Outcome measures

Outcome measures	Pre rehabilitation	Post rehabilitation
Manual muscle test upper limb	2/5	4/5
Manual muscle test lower limb	2/5	4/5
Barthel index	30/100	86/100
Hughes functional grading scale	Grade 3	Grade 0
Berg balance scale	32/56	48/56
Functional independence measure	89/126	119/126

## Discussion

Periodic paralysis can take many different forms, but the most common ones are related to abnormalities in metabolism and electrolyte balance. It is most common in hypoKPP, occurring once per 100,000 persons [16-18]. The primary feature of the illness is a sudden onset of weakness, which can range in severity from mild weakness that goes away quickly to severe weakness that could lead to respiratory failures. There is some variation in the disorder's clinical features. Attacks can be brought on by specific medications, such as beta-blockers or steroids, as well as stressors, such as illness or tiredness. The main cause of the inherited autosomal dominant primary hypoKPP is cold, high-carbohydrate diets, and hard labour, none of which were present in our case. Abuse of diuretics, hyperthyroidism, renal tubular acidosis, hyperaldosteronism, and Bartter syndrome have all been linked to secondary hypoKPP. Since his urine pH was normal and he did not have hyperchloremia, the possibility of renal tubular acidosis was ruled out [19].

Even though each case has a unique differential diagnosis, it appears to be a distinct case when the patient presents with a physical examination showing a loss in muscle power. The most serious conditions that need to be ruled out are paralysis and stroke, which can result in nerve compression. A thorough medical history that takes into account the onset, severity, and distribution of symptoms is necessary for the diagnosis of these disorders. Ion channels that carry calcium and sodium ions are responsible for both low potassium and muscle dysfunction. A patient may appear healthy in terms of potassium and strength, making diagnosis difficult in between paralytic episodes. Although there were no overt signs of hyperthyroidism, the patient had acute onset paralysis and obviously abnormal potassium and TSH levels [20]. This paralysis disappeared after taking medicine and receiving treatments. The patient's hemodynamically stable vital signs at the time included normal blood pressure, heart rate, and breathing rate. Trans-tubular potassium concentration gradient and potassium-creatinine ratio measurements could be used to rapidly and reliably distinguish between hypoKPP and non-hypoKPP. Patients diagnosed with hypoKPP should only take minimal supplements of potassium chloride to prevent rebound hyperkalemia [21]. When a patient presents with sudden weakness or paralysis, periodic paralysis should be taken into consideration, particularly if the patient has no significant risk factors for stroke or other medical conditions. To prevent paralysis from persisting or returning, it is imperative to treat the underlying cause as soon as possible [22].

## Conclusions

This case report concludes that physiotherapy plays a vital role in the management of hypoKPP since there is no established physiotherapy regimen for impairments specific to a given disease; thus, we attempted to manage hypoKPP by improving muscular strength and functional activities. Physiotherapy can benefit patients in a variety of ways, including improving their physical abilities and preventing disease adverse effects. By improving physical outcomes of patients and avoiding complications and recurrence, rehabilitation can improve the quality of life of patients with such disease.

## References

[1] Latorre R, Purroy F (2020). Hypokalemic periodic paralysis: a systematic review of published case reports [Article in Spanish]. Rev Neurol.

[2] Levitt JO (2008). Practical aspects in the management of hypokalemic periodic paralysis. J Transl Med.

[3] Finsterer J (2008). Primary periodic paralyses. Acta Neurol Scand.

[4] Ke Q, Luo B, Qi M, Du Y, Wu W (2013). Gender differences in penetrance and phenotype in hypokalemic periodic paralysis. Muscle Nerve.

[5] Statland JM, Fontaine B, Hanna MG (2018). Review of the diagnosis and treatment of periodic paralysis. Muscle Nerve.

[6] Fontaine B (2008). Periodic paralysis. Adv Genet.

[7] Clausen T (2010). Hormonal and pharmacological modification of plasma potassium homeostasis. Fundam Clin Pharmacol.

[8] Welland NL, Hæstad H, Fossmo HL, Giltvedt K, Ørstavik K, Nordstrøm M (2021). The role of nutrition and physical activity as trigger factors of paralytic attacks in primary periodic paralysis. J Neuromuscul Dis.

[9] Abbas H, Kothari N, Bogra J (2012). Hypokalemic periodic paralysis. Natl J Maxillofac Surg.

[10] Cannon SC (2015). Channelopathies of skeletal muscle excitability. Compr Physiol.

[11] Griggs RC, Engel WK, Resnick JS (1970). Acetazolamide treatment of hypokalemic periodic paralysis. Prevention of attacks and improvement of persistent weakness. Ann Intern Med.

[12] Baig M, Juneja P, Baig N, Ray K (2021). A rare case of a hypokalemic periodic paralysis while undergoing treatment for Addison's disease. Chest.

[13] Horlings CG, van Engelen BG, Allum JH, Bloem BR (2008). A weak balance: the contribution of muscle weakness to postural instability and falls. Nat Clin Pract Neurol.

[14] Vatwani A (2019). Pursed lip breathing exercise to reduce shortness of breath. Arch Phys Med Rehabil.

[15] Choi YH, Kim JD, Lee JH, Cha YJ (2019). Walking and balance ability gain from two types of gait intervention in adult patients with chronic hemiplegic stroke: a pilot study. Assist Technol.

[16] Osternig LR, Robertson R, Troxel R, Hansen P (1987). Muscle activation during proprioceptive neuromuscular facilitation (PNF) stretching techniques. Am J Phys Med.

[17] Victoria GD, Carmen EV, Alexandru S, Antoanela O, Florin C, Daniel D (2013). The PNF (proprioceptive neuromuscular facilitation) stretching technique - a brief review. Science, Movement and Health.

[18] Kaple N, Harjpal P, Samal SS (2022). Neuro-physiotherapy regimen to enhance the functional performance of a hemiplegic patient following brain tumor resection: a case report. Cureus.

[19] Meregildo-Rodríguez ED, Failoc-Rojas VE (2018). Case report: recurrent hypokalemic periodic paralysis associated with distal renal tubular acidosis (type 1) and hypothyroidism secondary to Hashimoto's thyroiditis. F1000Res.

[20] Ito N, Ihara K, Kamoda T (2015). Autosomal dominant distal renal tubular acidosis caused by a mutation in the anion exchanger 1 gene in a Japanese family. CEN Case Rep.

[21] Lin SH, Lin YF, Chen DT, Chu P, Hsu CW, Halperin ML (2004). Laboratory tests to determine the cause of hypokalemia and paralysis. Arch Intern Med.

[22] Cavel-Greant D, Lehmann-Horn F, Jurkat-Rott K (2012). The impact of permanent muscle weakness on quality of life in periodic paralysis: a survey of 66 patients. Acta Myol.

